# The Role of Placental Homeobox Genes in Human Fetal Growth Restriction

**DOI:** 10.1155/2011/548171

**Published:** 2011-04-12

**Authors:** Padma Murthi, Gayathri Rajaraman, Shaun Patrick Brennecke, Bill Kalionis

**Affiliations:** ^1^Department of Obstetrics and Gynaecology, The University of Melbourne, Melbourne, Victoria 3010, Australia; ^2^Pregnancy Research Centre, Department of Perinatal Medicine, The Royal Women's Hospital, Parkville, Victoria 3052, Australia; ^3^Monash Institute of Medical Research, Clayton, Victoria 3168, Australia

## Abstract

Fetal growth restriction (FGR) is an adverse pregnancy outcome associated with significant perinatal and paediatric morbidity and mortality, and an increased risk of chronic disease later in adult life. One of the key causes of adverse pregnancy outcome is fetal growth restriction (FGR). While a number of maternal, fetal, and environmental factors are known causes of FGR, the majority of FGR cases remain idiopathic. These idiopathic FGR pregnancies are frequently associated with placental insufficiency, possibly as a result of placental maldevelopment. Understanding the molecular mechanisms of abnormal placental development in idiopathic FGR is, therefore, of increasing importance. Here, we review our understanding of transcriptional control of normal placental development and abnormal placental development associated with human idiopathic FGR. We also assess the potential for understanding transcriptional control as a means for revealing new molecular targets for the detection, diagnosis, and clinical management of idiopathic FGR.

## 1. Introduction

### 1.1. Fetal Growth Restriction

The regulation of fetal growth is multifactorial and complex. Normal fetal growth is determined by the genetically predetermined growth potential and further modulated by maternal, fetal, placental, and environmental factors [1]. Fetal growth restriction (FGR), also known as intrauterine growth restriction (IUGR), is a failure of the fetus to reach its full growth potential for gestation age. FGR is commonly defined as a birth weight of less than the 10th percentile for gestation, together with evidence of fetal health compromise such as oligohydramnios and asymmetric fetal growth involving an increased head to abdominal circumference ratio. Evidence of such underlying pathology allows clinicians to discriminate between FGR and healthy small for gestation age (SGA) babies that are otherwise normal. FGR is associated with an increased risk of perinatal complications such as prematurity [2], stillbirth [2–5], neonatal morbidity [5, 6], and mortality [5, 6]. Adverse outcomes for FGR neonates include impaired neuropsychological development [7, 8] leading to reduced intelligence quotients [9, 10]. While FGR can be attributed to obvious fetal (e.g., chromosomal abnormalities), placental (e.g., obvious infarcts), maternal (e.g., tobacco smoking), and environmental factors (e.g., viral infections), about 70% of cases do not have a known cause and are termed idiopathic FGR. Idiopathic FGR is frequently associated with placental insufficiency [11]. Cordocentesis studies (sampling of umbilical fetal arterial or venous blood) show features consistent with chronically inadequate transplacental oxygen exchange between the mother and FGR fetus [11]. Clinical features of idiopathic FGR pregnancies include abnormal umbilical artery Doppler velocimetry [12], oligohydramnios [13], and asymmetric fetal growth [14]. 

### 1.2. Pathology of Placental Dysfunction in FGR

Typically, the placentae in idiopathic FGR are smaller than their gestation age-matched controls [15], and they show obvious morphological defects. Macroscopic placental lesions [12] are frequently evident, whilst microscopic defects such as reduced trophoblast proliferation and abnormal villous vasculature with shorter, less branched terminal villi [16] are also observed. Another significant functional defect is uteroplacental ischemia due to failure of the placental extravillous cytotrophoblast cells to effectively carry out the critical processes of invasion, transformation, and remodeling of the spiral arteries in the maternal decidua [17]. 

At the cellular level, trophoblast function is modulated in an autocrine/paracrine manner by growth factors, their binding proteins, and extracellular matrix components of the placenta (reviewed in [18, 19]). This modulation of trophoblast cell function involves various extracellular signals, signalling molecules, and consequent receptor activation in the signalling pathway. Disruption of various important signalling pathways is observed in placental pathologies that are associated with abnormal trophoblast function [20].

A consequence of altered placental function in idiopathic FGR is reduced transfer of oxygen, nutrients, and growth factors to the fetus, which restricts fetal growth [21]. The changes observed in FGR placentae are consistent with early developmental defects [17], but the developmental genes involved and their molecular mechanism of action are not known. Several longitudinal studies have demonstrated a possible causative role for genetic and familial factors, as yet unidentified, in human FGR [22, 23]. 

Current knowledge of the molecular mechanisms of FGR is limited. Various attempts to understand the molecular basis of FGR using microarray and proteomics approaches have revealed significant differences between FGR and uncomplicated control [24–26] on term placentae and have shed little light on the regulatory mechanisms that underlie the early events leading to idiopathic FGR. Murine model systems, particularly those amenable to genetic manipulation, are therefore of crucial importance in revealing potentially important regulatory genes that may play a role in the early stages of human FGR. In many animal model systems, early developmental stages are controlled at the level of transcription factors. 

## 2. Transcriptional Control of Placental Development

Growth factors and signalling molecules represent the cue to which a cell responds by either maintaining or altering its state of differentiation [27]. However, it is the transcription factors, located within the cell nucleus, which determine how this cue is interpreted and what the cellular response will be. Transcription factors achieve this by regulating expression of their target genes within the cell. A large number of different transcription factors play essential roles in cellular development and differentiation of various cell types, including the trophoblast cell type in the placenta [28, 29]. Transcription factors are categorised into a few large families such as the zinc finger, leucine zipper, helix-loop-helix, helix-turn-helix, and homeobox genes [30, 31]. 

## 3. Homeobox Genes

Homeobox genes (also known as homeotic genes) were originally discovered in the fruit fly *Drosophila,* where they act as transcriptional regulators to control embryonic morphogenesis (reviewed in [32–34]). These genes contain a highly conserved 180 base pair homeobox sequence, which encodes a 60 amino acid homeodomain. Structural analyses have shown that the homeodomain consists of an evolutionarily conserved helix-turn-helix motif that binds to the DNA. The specificity of this binding allows homeodomain proteins to activate or repress the expression of batteries of downstream target genes [35]. 

Most important is that homeobox genes are directly or indirectly involved in a variety of developmental disorders, diseases, and cancers (reviewed in [36]). Homeobox genes are subdivided into the “clustered” homeobox genes known as “HOX” genes, the “nonclustered” divergent or orphan HOX-like genes, as well as several distinct classes of atypical homeodomain containing genes. The HOX family plays a fundamental role in the embryonic morphogenesis and were identified in mammals and vertebrates based on their sequence homology to the genes of the *Drosophila* HOM-C [37, 38]. In mice and humans, the HOX complex is comprised of 39 genes that are arranged into four separate chromosomal clusters designated HOX A, B, C, and D [39, 40]. 

Homeobox genes are grouped together into various subfamilies based on a variety of criteria such as their functional and structural characteristics, and these subfamilies of homeobox genes are essential for the control of specific aspects of cellular growth and differentiation [28, 29, 41]. Evidence for the deregulation of certain homeobox genes in cancer and other diseases provides support for the idea that homeobox genes are vital for normal mammalian development. Furthermore, characterisation of such homeobox genes may lead to a greater understanding of the developmental mechanisms, which are disrupted in a variety of disease states. There is evidence that normal homeobox gene expression can be altered during a diseased state, such as decreased expression of Cdx2 in the intestinal epithelium of patients with colorectal cancers and decreased Meox2 expression in brain endothelial cells of patients affected by Alzheimer's disease [36, 42]. Thus, homeobox genes could be used as disease markers or potential therapeutic targets of diseases, such as cancer, diabetic wound healing, lymphedema, Alzheimer's disease, and stroke due to atherosclerosis [43–45]. 

Homeobox gene mutations have also been shown to cause human congenital disorders such as Waardenburg's syndrome type 1 [46, 47] and Aniridia [48]. The homeobox gene HuP2 has been found to be mutated in patients with Waardenburg's syndrome [46, 47], and the congenital eye disorder Aniridia caused by a mutation in the homeobox gene designated AN [48]. 

The clustered homeobox genes, known as HOX, play a fundamental role in embryological morphogenesis. HOX gene mutations are implicated in various human malformations such as hand-foot-genital syndrome, Mowat-Wilson Syndrome, and Duanes Retraction Syndrome (reviewed in [36]). There is also an association between mutation in HOX genes and autism spectrum disorders [49]. More recently, the Aristaless-related homeobox gene, ARX, was found to be associated with both X-linked mental retardation and epilepsy [50, 51]. 

Mouse knockouts have also provided genetic proof that homeobox genes regulate embryonic organogenesis and morphogenesis [52–54]. For example, targeted disruption of the *Hlx* homeobox gene (the homolog of human *HLX*) in the mouse shows that *Hlx* plays a fundamental role in visceral organogenesis [55]. Studies have demonstrated that *Hlx* mutant mice resulted in developing gut and liver diverticulum defects. In addition, *Hlx* mutation also showed a defect in cell proliferation and resulted in embryonic death due to liver failure [55]. Furthermore, *Hlx* is expressed in mesenchymal cell types during organogenesis in the mouse placenta [56]. Additionally, recent studies from our laboratory have confirmed that placental morphology is severely affected in *Hlx* mutant mice (Murthi et al. unpublished data). 

### 3.1. Homeobox Genes in Murine Placental Development

Given the highly important role of homeobox genes in embryonic and adult development, it is not surprising that homeobox genes also play major roles in controlling extraembryonic development of the placenta. Homeobox genes regulate mouse placental cell functions and targeted gene mutations of homeobox genes in the mouse produce FGR-like effects. For example, homeobox gene mouse mutants, *Esx1* and *Dlx3*, produce FGR-like effects in mice including restricted fetal growth and placental defects [57, 58]. *Esx1* expression is restricted to the placenta and is not expressed in the embryo. Thus, in the *Esx1* mutant mouse, altered placental function is the cause of restricted fetal growth. *Dlx3* and *Esx1* mutant mice show specific defects in the labyrinthine trophoblast of the chorioallantoic placenta [57, 58]. In addition, the 3 beta-hydroxysteroid dehydrogenase gene (*3*β*-HSD*), which is important for the biosynthesis of all active steroid hormones, is a target of the *Dlx3* homeobox gene in the mouse [59]. Therefore, homeobox genes control important trophoblast functions in the mouse placenta. 

The homeobox gene *Cdx2* is expressed in the embryonic trophectoderm and in the spongiotrophoblast component of the placenta at later stages of development and is implicated in the patterning of the extraembryonic membranes of the mouse placenta [60]. The finding that *Cdx2* homozygous null mutant mice die between 3.5 and 5.5 days as a consequence of failed implantation suggests that this homeobox gene may play a role in controlling trophoblast differentiation [61]. 

The placenta specific–homeobox gene (*Psx*) also affects mouse placental development. The *Psx* transcript was first detected at embryonic day 8.5 and expression persisted until birth. *Psx* mRNA is expressed in extraembryonic tissues, mainly in the placenta, but not in the fetus [62]. Further studies have shown that the *Psx* homeobox gene plays a unique role in the function of differentiated trophoblast cells in the murine placenta [63]. 

Mouse homeobox gene knockouts have also provided evidence that homeobox genes regulate vascular development and angiogenesis in the mouse placenta (reviewed in [29, 41, 64]). Therefore, in animal model systems, homeobox genes control trophoblast and endothelial cell functions during placental development. 

### 3.2. A Strategy for Understanding Transcriptional Control in Normal and FGR-Affected Placentae

Our strategy for understanding the molecular mechanisms of placental function in normal and FGR-affected human placentae involved (i) determining the spatiotemporal expression pattern of homeobox genes during placental development that have an “evolutionary history” of regulating cell fate decisions during embryonic or adult development, (ii) determining whether specific homeobox gene expression levels were changed in FGR-affected placentae compared with gestation matched controls, (iii) creating *in vitro* models of placental cultured cells that “mimic” homeogox gene expression changes observed on FGR by the use of loss- or gain-of function phenotypes using RNA interference systems or gene overexpression plasmids, and (iv) defining the biological functions of the target genes using *in vitro* models. These approaches have been proven very successful in identifying transcriptional control of endocrine functions during mouse placental development (reviewed in [28, 29]). Therefore, identification of the homeobox target genes in specialised cell types of the human placenta can reveal the molecular pathways responsible for important placental cell functions. These pathways may be affected in FGR. Using this novel approach, more recent studies in our laboratory have described a potential role for transcriptional control of homeobox gene HLX in the human placental trophoblast cells. In the following section, we will summarise our current understanding of homeobox gene HLX regulation in human placental development, more specifically to human extravillous trophoblast function, as well as give insights into novel mechanisms of trophoblast dysfunction observed in FGR-affected pregnancies.

**(i) Spatiotemporal Expression Patterns of Homeobox Genes in the Placenta**

Studies in the human placenta have focused mainly on identifying homeobox genes expressed in the normal placenta [65, 66], and those showing altered expression in trophoblastic cancers [67]. The homeobox genes we and others have identified to be of potential importance in the human placenta are *DLX3 *[59, 68, 69]*, DLX4 *[70–72],* MSX2 *and* GAX * [70], * ESX1L *[58, 73], and *HLX * [74–77]. These genes are potential candidates for regulating epithelial-mesenchymal cell interactions in the human placenta. These genes are potential candidates for regulating epithelial-mesenchymal cell interactions in the human placenta. These genes are also expressed in the embryo and play major roles in embryonic development [78, 79]. Microarray expression profiling of placental trophoblast and endothelial cells revealed that novel placental homeobox genes *TGIF, MEIS2E, LIM2*, and *SMAP31-12 *are also highly expressed in trophoblast cells (Murthi et al. unpublished data).

Few functional studies have been carried out on human placental homeobox genes. One limited study reported that the inactivation of homeobox gene *DLX4 *resulted in altered rates of trophoblast cell apoptosis [72]. Homeobox gene *DLX3 *regulates the expression of the alpha subunit of hCG [59] and of 3-**β**HSD [69], both of which are important for placental trophoblast function.

### 3.3. Homeobox Genes in Human Placental Endothelial Cells

Knowledge of homeobox genes in human endothelial cells comes primarily from studies in the cardiovascular system employing cell culture models such as human umbilical vein endothelial cells (HUVEC). Homeobox genes are critical regulators of cardiovasculature development [80]. *GAX* is a negative regulator of angiogenesis [81]. *HOXB3* promotes invasive behaviour of endothelial cells in response to angiogenic stimulation [54], whereas *HOXD3* promotes capillary morphogenesis [82]. In HUVEC stimulated with VEGF, *HEX* acts as a negative regulator of angiogenesis [83]. Also in HUVEC, *GAX* is an inhibitor of endothelial cell activation in response to growth factors and tube formation [53]. 

Previous studies from our laboratory have demonstrated the expression of homeobox genes *HLX, DLX3, DLX4, MSX2*, and *GAX *in placental endothelial cells, and we showed that novel placental homeobox genes, such as*TLX1, TLX2, TGIF, HEX, PHOX1, MEIS2, HOXB7, *and *LIM6 *were also expressed in placental endothelial cells [84]. Our findings have highlighted the potential importance of these genes in the fundamental process of placental angiogenesis. Clearly, homeobox genes are important regulators of endothelial cell functions in the embryo and adult but their role in placental endothelial cells is yet to be determined.

**(ii) Homeobox Gene Expression Levels Are Changed in FGR Placentae Compared with Gestation-Matched Controls**

Previous studies from our laboratory determined the expression levels of several homeobox genes in a clinically well-defined idiopathic FGR-affected placentae and gestation-matched controls [71, 73, 75]. The cohort of FGR-affected pregnancies that was employed was carefully defined in clinical terms and represented the severe end of spectrum of idiopathic FGR. The general inclusion criterion for FGR cases was a birth weight less than the 10th centile for gestation age, using Australian growth charts. FGR cases were classified as idiopathic if there was evidence of an underlying pathology, judged by the presence of at least two of the following antenatal ultrasound diagnostic criteria: abnormal umbilical artery Doppler flow velocimetry, oligohydramnios as determined by amniotic fluid index (AFI) <7, or asymmetric growth of the fetus as measured from the HC (head circumference) to AC (abdominal circumference) ratio (>1.2). Fetuses showed reduced growth by the late second and early third trimester. Reduced villous tree elaboration, diminished surface area of the placenta, and abnormal end-diastolic blood flow in the umbilical artery are characteristic of pregnancies with severely growth-restricted infants [15, 16]. Homeobox genes *HLX* [75] and *ESX1L* [73] showed decreased expression in FGR-affected placentae compared with matched controls.

The pattern of normal human fetal growth is complex. Increases in the rates of fetal weight gain and length increase are not parallel throughout pregnancy. Evidence suggests that the maximal growth rate for length is seen in the second trimester, whereas the maximal rate of weight gain is early in the third trimester [75, 83]. Guihard-Costa et al. [85] in a longitudinal study of human fetal growth have reported a linear growth rate until 26 weeks and, thereafter, the growth rate decreased. In our studies, a rapid decline in the levels of both *HLX *and *ESX1L *expression was observed from 27-week gestation, which may correspond to the decline in the growth rate of the fetus seen in the third trimester [81, 86].

Our studies represented the most comprehensive and extensive analyses of homeobox genes in placental pathologies undertaken. However, homeobox gene *DLX4* showed increased expression [71] in FGR-affected placentae. Our observation of altered homeobox gene expression levels, that is, decreased (*HLX*) or increased (*DLX4*) expression in FGR-placentae compared with gestation matched controls, prompted us to identify the downstream target genes which would be affected by changed homeobox gene levels.

**(iii) Creating In Vitro Models of Placental Cultured Cells That “Mimic” Homeobox Gene Expression Changes Observed on FGR**

Homeobox gene *HLX* is the most characterised in the human placenta. The *HLX *gene (also known as *HLX1*, H2.0-like homeobox or *HB24*; OMIM 142995) is a member of the homeobox family of genes, with homology to the *Drosophila* homeobox gene *H2.0*. A comparison of HLX orthologs in human and mouse showed that the genes share similar organization, with four exons and three introns and 85.4% identity between the human and mouse proteins, suggesting a similar function in both species [87]. The *HLX *homeobox gene was shown to have high expression in haematopoietic progenitor cells, and lower expression levels in activated lymphocytes [88].

Our studies demonstrated that* HLX* is expressed primarily in the proliferating cytotrophoblast cell types in early placental development and suggested that reduced levels of *HLX* are required for cytotrophoblast differentiation and that dysregulation of *HLX* may result in aberrant cytotrophoblast proliferation and differentiation, contributing to placental pathologies [74].

Furthermore, to identify the functional role of reduced HLX levels observed in FGR, we simulated reduced expression levels in extravillous trophoblast derived cell lines SGHPL4 and HTR8-SV neousing short-interference RNA (siRNA) specific for HLX. These two transformed trophoblast-derived cell lines are well-characterized first trimester-derived human extravillous cytotrophoblast cell lines and are capable of proliferation, migration, and invasion. The results from this study were not cell line specific, since consistent effects were seen in both the cell lines tested.

Our findings provided evidence of *HLX* regulation by cytokines, CSF-1, and growth factors such as HGF, and established that *HLX* is an important regulator for signal transduction mediated proliferation and migration of human extravillous trophoblast cells [76, 77] suggesting that *HLX *may be of pathological significance.

**(iv) Defining the Biological Functions of the Target Genes Using In Vitro Models**

Understanding the precise regulatory mechanisms through which homeobox genes achieve molecular control during placental development requires the identification of target genes within the downstream developmental pathways.

Genes involved in regulating cellular mechanisms such as mitotic rate, cell-cell adhesion, and cell migration during morphogenesis have been identified as target genes for many homeobox genes [89–99]. Thus, homeobox genes act as “master regulators” of development and control transcription by binding to regulatory elements in the promoter regions of target genes [38].

In the last two decades, the purification, cloning, and characterisation of several homeobox transcription factors, together with transgenic mouse models, have increased our knowledge of the molecular basis of placental development. Whilst loss-of-function studies in the mouse model clearly demonstrate that homeobox genes such as *Cdx2, Cdx4, Hoxa13*, and *dlx3* are critical for murine placental development [60, 68, 100], the target genes regulated by these homeobox genes have not been investigated in either the murine or human placenta.

### 3.4. Identification of HLX Target Genes

Previous studies showed that inhibition of *HLX* by antisense oligonucleotide methods impaired CD34+ bone marrow cell proliferation in response to stimulation by cytokines, whilst inducing differentiation of these cells. Moreover, *HLX* inhibition also reduced the levels of *c-myc*, *c-fos*, *cyclin B*, and *p*34^cdc2^ mRNA expression [88]. These cell cycle regulatory genes were predicted to be involved in the function of trophoblast cells [101]. By using siRNA-mediated inactivation of *HLX* approach, we investigated the mechanisms by which *HLX* mediates extravillous trophoblast function in normal and FGR-affected placentae. We used siRNA in trophoblast *in vitro *models such as SGHPL-4 and HTR-8/SVneo and detected changes in gene expression using pathway-specific low density PCR arrays for MAP- (mitogen-activated signaling-)kinase signaling pathways. The downstream target genes of *HLX* were identified as *RB1, MYC, EGR1, CDKN1C, ELK1, CCNB1*, and *JUN*. These findings were further validated suggesting the observations were not only consistent in two independent trophoblast cell lines, SGHPL-4 and HTR-8/SVneo, but was also reflected in FGR-affected human placental tissue. Most importantly, we identified four *HLX* downstream target genes *CCNB1, MYC, CDKN1C*, and *JUN,* which were previously identified as *HLX *target genes in haematopoietic progenitor cells [88] as targets of* HLX* in cultured trophoblast cells. Thus, *HLX* homeobox gene targets cell cycle regulatory genes in two independent cell types. 

In the following section, we have described further analyses of candidate downstream target genes of *HLX* and their level of expression and potential contribution to functional abnormalities observed in FGR-affected placentae. 

Retinoblastoma-1 (*RB1*, also known as *Rb*) is a tumor suppressor gene that was first discovered in genetic studies of hereditary retinoblastoma [102]. *RB1* also has a role in other cancers including osteosarcoma and plays an important role in regulating cell proliferation and differentiation [103]. The product of the *RB1* gene is a nuclear phosphoprotein that may act as an inhibitor of cell proliferation [104]. Additionally, Schubert et al. [105] have demonstrated that *RB1* is a downstream target of the GCMa/Gcm1 transcription factor in the mouse placenta. Therefore, *RB1* has been shown to be a direct target of transcription factors. In mice, the constitutive knockout of *RB1* causes embryonic lethality resulting from defects in placental function [106] reviewed in [107]. Wu and coworkers [106] have demonstrated that reduction of *RB1* gene expression in the mouse model system resulted in excessive proliferation of trophoblast cells and a severe disruption of the normal labyrinth architecture in the placenta. This was accompanied by a decrease in vascularisation and a reduction in placental transport function and ultimately embryonic death [106].

Our findings demonstrated that *RB1* is a direct or indirect downstream target of the homeobox gene *HLX *in cultured human trophoblast cells [108]. Furthermore, *RB1* is expressed in the proximal region of proliferating trophoblast cells in the trophoblast cell column [109], where *HLX* is also expressed [74]. This provided supporting evidence *HLX* may act as a regulator of *RB1* in trophoblast cells and that *HLX*-mediated *RB1* expression in trophoblast cells may reduce trophoblast proliferation. We also observed that *RB1* showed the highest relative increase in expression levels in FGR-affected placentae compared with control placentae [108]. These data suggest *RB1* is a negative regulator of cell proliferation and that increased *RB1* expression levels in FGR may reduce trophoblast proliferation and result in a fewer number of trophoblast cells available to migrate and invade into the maternal decidua. This reduction in trophoblast proliferation may also lead to the shallow, inadequate remodeling of the maternal spiral arteries associated with FGR. 

*MYC* is a proto-oncogene that is overexpressed in a wide range of human cancers. This cell cycle regulator gene is part of the postreceptor intracellular signaling pathway for regulation of cell proliferation by growth factors [110]. Depending on the cellular context, *MYC* proteins induce either cell proliferation or apoptosis and they require cooperation with other oncoproteins and inhibition of apoptotic pathways to transform cells [111]. Previous studies have determined that *MYC* is expressed in the actively proliferating extravillous trophoblast cells of the human placenta [112–114], where we have shown *HLX* to be highly expressed [74]. Results from our study demonstrated that *MYC* mRNA expression was significantly increased with *HLX* inactivation in cultured trophoblast cells, suggesting that *MYC* is a direct or indirect downstream target gene of *HLX* [108]. 

Targeted disruption of *c-myc* gene (homolog of *MYC*) in the mouse model system resulted in severe placental defects including morphological abnormalities [115]. Several embryonic developmental defects were also reported including abnormalities in the heart, liver, and neural tube formation. More importantly, embryonic death was also observed in *c-myc* knock-out mice due to placental insufficiency [115]. Our findings showed that *MYC* expression was significantly increased in FGR-affected human placentae, consistent with the increase in *MYC* expression in *HLX* inactivated cultured trophoblast cells [108]. This suggested that *MYC*, as a downstream target gene of *HLX*, is a molecular target associated with idiopathic human FGR. Therefore, *HLX*-mediated increase in *MYC* expression may contribute to increased apoptosis that is frequently associated with FGR.

*CDKN1C *(p57/kip2) is a member of the *CIP/KIP* family of cyclin-dependent kianse inhibitors and has been shown to inhibit several cyclin-dependent kinase kinase/cyclin complexes and is a regulator of cell proliferation [116]. Mutations of *CDKN1C* are implicated in sporadic cancers and Beckwith-Wiedemann syndrome suggesting that it is a tumor suppressor candidate. Larson et al. [117] have suggested that decreased *CDKN1C* expression may be involved in human breast carcinogenesis *in vivo*. *CDKN1C* has recently been recognized as a maternally imprinted gene, supporting its role for genomic imprinting in the regulation of embryonic implantation and development and placental growth, as well as in the pathogenesis of proliferative trophoblastic diseases [118]. In the normal placenta, strong nuclear *CDKN1C* expression was observed in extravillous trophoblast, cytotrophoblast, and implantation-site interstitial trophoblast, but was absent in syncytiotrophoblast [118]. This expression of* CDKN1C* in the human placenta is consistent with the expression pattern of *HLX *from our study [74]. 

Studies have shown that targeted disruption of *CDKN1C* in the mouse model system results in severe placental defects [119]. *CDKN1C *knock-out mice have displayed an array of pre-eclampsia symptoms, including placental abnormalities, hypertension, proteinuria, and premature labour [119]. Results from our own findings showed that *CDKN1C* expression is significantly reduced in cultured trophoblast cells, therefore, is a direct or indirect target candidate gene of *HLX *in cultured trophoblast cells [108]. This suggests that *HLX*-mediated reduction of *CDKN1C* expression may reduce trophoblast proliferation. Further confirmation of *CDKN1C* mRNA expression in FGR-affected human placentae, also show a significant decrease in human idiopathic FGR compared with gestation-matched controls. 

*ELK1*, as a member of the *ETS* family, acts as a transcriptional factor for the *MET* gene [120], which is expressed in placental cytotrophoblasts [121]. As with *HLX*, *ELK1* is also expressed in human extravillous trophoblast cells [122] and is suggested to play a role in the regulation of cell proliferation and migration [123]. *ETS* transcription factors are also critical for human uterine decidualisation [124]. Human decidual fibroblasts expressed significantly less mRNA for the decidualisation markers prolactin, IGFBP-I, EBAF, TIMP3, decorin, and laminin in the presence of an antisense oligonucleotide that blocks the translation of *ETS* mRNA when compared with decidual fibroblast cells exposed to a control oligonucleotide [124]. 

Given our previous findings of *HLX* expression in the human placenta [74] and in the importance of *HLX* in trophoblast proliferation [77] and migration [76], *ELK1* is a potential target gene of *HLX* in the control of trophoblast cell proliferation and migration. Consistently, results from our observation showed that *ELK1* expression was also significantly decreased in FGR-affected human placentae. Therefore, the cell culture model where siRNA-mediated reduction of *HLX* reduces *ELK1 *was consistent with decreased levels of *HLX* and decreased levels of *ELK1* in human FGR.

*CCNB1* is a regulatory gene expressed predominantly during the G2/M phase of the cell cycle, functionally involved in cell mitosis. Studies have shown expression of *CCNB1* in the villous trophoblast and the extravillous trophoblast cell types of the human placenta [125, 126], which also correlates with placental *HLX* expression from our findings [74]. This current study showed that *CCNB1* mRNA expression was significantly reduced in cultured trophoblast cells with *HLX* inactivation, suggesting that *CCNB1* to be a downstream target gene of *HLX*. *CCNB1* expression was also significantly reduced in FGR-affected placentae compared with controls. Therefore, our findings showed that the cell culture model was consistent with the observed changes seen in *HLX* levels in FGR and changes in *CCNB1* levels and suggested a causative role between reduced *HLX* levels and reduced *CCNB1* levels in FGR.

*JUN *is a member of the *AP-1* family of transcription factors and is implicated as a key regulator of human extravillous trophoblast proliferation, invasion, and differentiation [127]. Not surprisingly, *JUN* is also strongly expressed in the highly proliferative extravillous trophoblast cells of the human placenta [127], consistent with *HLX* expression in the human placenta [74]. *JUN* plays a key role in coordinating steroid hormone actions in a variety of tissues [128] and is induced by the steroid hormone oestrogen in the human endometrium. Salmi and Rutanen [129] have demonstrated a strong expression of *JUN* in human proliferative endometrium and that *JUN* expression is decreased in the human decidua throughout pregnancy [130]. 

Our own findings showed significantly decreased *JUN *expression with *HLX *gene reduction in cultured trophoblast cells. This suggests, as a downstream target of *HLX*, *JUN *is regulated by *HLX*, either directly or indirectly, in order to affect proliferation, invasion, and differentiation of extravillous trophoblasts. Consistently, results showed that *JUN *expression was also significantly decreased in FGR-affected human placentae compared with control placentae. Therefore, decreased *JUN *expression, either directly or indirectly by *HLX*, may result in anomalous trophoblast functions associated with FGR. Furthermore, *HLX*-mediated *JUN *dysregulation of trophoblast differentiation and invasion can lead to abnormal spiral artery remodeling by endovascular trophoblasts, as these endovascular trophoblasts need to differentiate from cytotrophoblasts and invade the maternal spiral arteries for enhanced blood flow during pregnancy [16].

Thus, the candidate downstream target genes of a homeobox gene, *HLX*, are significantly altered in human idiopathic FGR-affected placentae, compared with gestation-matched controls. Most importantly, the findings of our own study demonstrated that *in vitro* models for siRNA-mediated knockdown of *HLX* expression in placental trophoblast cells show consistent changes to those observed in human FGR where *HLX* levels are reduced. These results suggest that reduced levels of *HLX* seen in FGR cause direct or indirect effects on target genes that have been shown to be altered in FGR. Therefore, reduced *HLX* levels directly or indirectly cause gene expression changes in targets that have deleterious effects on trophoblast function. 

## 4. Conclusions and Future Directions

Rapid progress in understanding placental development, and its regulatory molecules has been achieved in the last decade. Placental development, as does development of other embryonic organs, progresses through many step and some key regulators have now been identified. However, more work is required to complete the analyses of both molecular and cellular events on various human placental cell types remains. Our current understanding of how homeobox genes regulate trophoblast functions suggest that important aspects of regulation are conserved between the extraembryonic placenta and embryonic morphogenetic and differentiation events. 

It is evident from our findings that homeobox genes such as HLX play a critical role in trophoblast function and involve molecular and cellular mechanisms that have been observed during differentiation and morphogenesis of the embryonic tissues. This may reflect the involvement of *HLX* in regulating the fundamental process of proliferation, the regulatory mechanisms of which are likely to be highly conserved. Studies of targets of other homeobox genes may reveal the regulation of more specialised placental cell functions.

The strategy we have employed has resulted in the identification of homeobox genes, which are expressed in normal placental development and that show altered expression in FGR. Functional assays following target gene inactivation in cultured cells reveal that homeobox genes control important functions in placental cells. The discovery of targets of homeobox genes has revealed genes, and pathways, not previously implicated in FGR. These target genes and pathways will be further assessed for their therapeutic and diagnostic potential in future. 
